# Three-dimensional crossbar arrays of self-rectifying Si/SiO_2_/Si memristors

**DOI:** 10.1038/ncomms15666

**Published:** 2017-06-05

**Authors:** Can Li, Lili Han, Hao Jiang, Moon-Hyung Jang, Peng Lin, Qing Wu, Mark Barnell, J. Joshua Yang, Huolin L. Xin, Qiangfei Xia

**Affiliations:** 1 Department of Electrical and Computer Engineering, University of Massachusetts, Amherst, Massachusetts 01003, USA; 2 Center for Functional Nanomaterials, Brookhaven National Laboratory, Upton, New York 11973, USA; 3 Air Force Research Laboratory, Information Directorate, Rome, New York 13441, USA

## Abstract

Memristors are promising building blocks for the next-generation memory and neuromorphic computing systems. Most memristors use materials that are incompatible with the silicon dominant complementary metal-oxide-semiconductor technology, and require external selectors in order for large memristor arrays to function properly. Here we demonstrate a fully foundry-compatible, all-silicon-based and self-rectifying memristor that negates the need for external selectors in large arrays. With a p-Si/SiO_2_/n-Si structure, our memristor exhibits repeatable unipolar resistance switching behaviour (10^5^ rectifying ratio, 10^4^ ON/OFF) and excellent retention at 300 °C. We further build three-dimensinal crossbar arrays (up to five layers of 100 nm memristors) using fluid-supported silicon membranes, and experimentally confirm the successful suppression of both intra- and inter-layer sneak path currents through the built-in diodes. The current work opens up opportunities for low-cost mass production of three-dimensional memristor arrays on large silicon and flexible substrates without increasing circuit complexity.

## Abstract (web summary)

Memristors are key structural units of complex memory and computing systems, yet most currently available memristors are based on materials that are not compatible with silicon technology. Here, the authors demonstrate a CMOS-compatible, self-rectifying memristor and arrays entirely based on p-Si/SiO_2_/n-Si.

Memristors are two-terminal devices that exhibit voltage and/or current actuated resistance switching behaviour with a signature of pinched hysteretic current–voltage (IV) loops^1^,^2^,^3^. With proved advantages in scalability^4^, switching speed^5^, power consumption^6^, endurance^7^ and three-dimensional (3D) stackability^8^,^9^,^10^, these devices have been proposed and demonstrated for applications such as non-volatile memory^9^,^11^, reconfigurable logic^8^, neuromorphic computing^12^,^13^ and radiofrequency switches^14^. A memristor is usually composed of two metallic electrodes that sandwich a layer of switchable material, among which transition metal oxides and perovskites have been the most popular choices to date^15^. Unfortunately, these materials and associated fabrication processes are costly and not fully compatible with the complementary metal-oxide-semiconductor (CMOS) platform. On the other hand, resistance switching phenomena in silicon oxide have been observed since 1960s and attracted revived interest recently because of their CMOS compatibility^15^,^16^,^17^,^18^,^19^,^20^,^21^,^22^. However, the device performance needs further optimization and the switching mechanism is still under intensive debate^16^,^17^, and devices and arrays made of all-silicon-based materials are yet to be demonstrated, let alone 3D stacking of the crossbar arrays.

Building high density, massively parallel memristor crossbar arrays is critical for most of the aforementioned applications. One of the prominent challenges to access an individual device in such an array is to solve the sneak path problem that leads to operational failure and high power consumption^3^. To address this issue, selector devices such as rectifying diodes^18^, nonlinear bidirectional selector^19^,^20^,^21^,^22^ and complementary resistive switches^23^,^24^ have been proposed and demonstrated to suppress the sneak path current through the unselected devices. Silicon-based selector devices^25^,^26^,^27^, such as diodes and transistors, provide promising solutions because of materials compatibility and process maturity. To fabricate these selector devices, epitaxy growth of Si diodes^28^,^29^,^30^, chemical vapour deposition of polysilicon diodes^26^ and amorphous silicon diodes have been employed. However, they rely on high temperatures for silicon crystallization and dopant activation, and hence are not necessarily compatible with back-end-of-line (BEOL) processes, especially for wearable electronics on flexible substrates that typical do not sustain elevated temperatures. On the other hand, Si membrane transfer technology based on transfer printing^31^,^32^, fluid-supported transfer^33^,^34^ has been proposed to build logic circuits on flexible substrate or photonic crystals, which showed great potential to overcome the difficulties in building 3D stackable and BEOL compatible Si-based selectors.

In this paper, we report an all-silicon-based memristor with a built-in rectifying selector that is fully compatible with CMOS platform. The device employs p- and n-type doped single crystalline silicon transferred from fluid-supported membranes as top and bottom electrodes and a thin layer of chemically produced silicon oxide as the switching layer^31^. The device exhibits repeatable self-rectifying resistive switching with high rectifying ratio (10^5^), high ON/OFF conductance ratio (10^4^) and long data retention at elevated temperature (>2 × 10^5^ s at 300 °C). We attribute the switching behaviour to the formation and rupture of a silicon-rich conductive filament inside the oxide, as suggested by current–voltage (IV) curve analysis and transmission electron microscopy (TEM) studies. The self-rectifying effect is due to the different doping types in the silicon electrodes that forms a self-assembled diode within each junction. We further construct 3D crossbar arrays of Si/SiO_2_/Si memristors (up to five layers of 100 nm × 100 nm memristor array) and demonstrate that the built-in selectors effectively alleviate both intra- and inter- layer sneak path problem. The all silicon-based materials and room-temperature fabrication process are fully compatible with CMOS technology, opening up opportunities for low-cost volume production of large arrays of memristor devices and hybrid memristor/CMOS circuits on a variety of substrates.

## Results

### Device fabrication and electrical characterization

**Figure 1:**
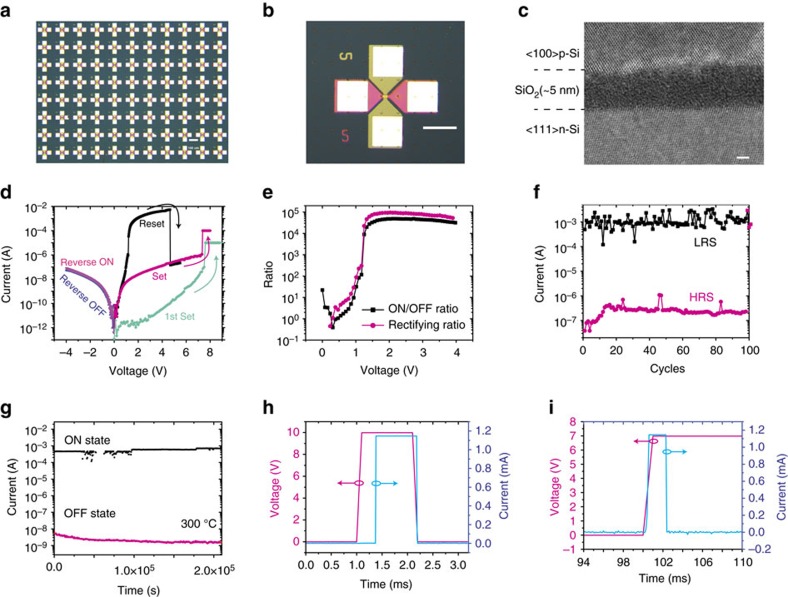
Images and electrical behaviour of p-Si/SiO_2_/n-Si memristors. (**a**) Top view of a 11 × 8 array of single cross-point devices with high fabrication yield. Scale bar, 100 μm. (**b**) Zoom-in image of one 5 μm × 5 μm cross-point device. Scale bar, 50 μm. (**c**) Cross-sectional TEM image for the vertical stack of the Si/SiO_2_/Si device, showing single crystalline structure for the top and bottom electrodes and the 5 nm amorphous SiO_2_ switching layer. Scale bar, 2 nm. (**d**) Typical unipolar resistive switching curves. The bias was applied on the p-Si top electrode while the n-Si bottom electrode was grounded. The RESET voltage was about 4.5 V while SET voltage was about 7.5 V (voltage drop on wires included). The reverse current was suppressed regardless of its state. The turquoise curve is the first SET with almost the same voltage as the following ones, indicating the formatting–free nature of the device. (**e**) The ON/OFF conductance ratio and rectifying ratio as a function of the bias voltage. The ON/OFF ratio was more than 10^4^ and rectifying ratio almost 10^5^ when bias voltage was larger than +1.5 V. (**f**) DC switching of 100 consecutive cycles. The current was read at +3 V. (**g**) Retention performance tested at 300 °C. The device maintained both states for more than 2 × 10^5^ s. (**h**,**i**) The transient resistive switching under electric pulses, which shows the current switching speed for **h**. SET and **i**. RESET process. The magenta curves are the applied voltage pulse and blue curves the current. The SET speed is 7.6 μs under 10 V, while the RESET is about 490 μs under 7 V. The switching speed is still under optimization, and a tradeoff between the speed and the voltage pulse amplitude is expected.

To fabricate the cross-point device, a p-type doped (dopant concentration *N*_A_∼10^20^ cm^**−**3^) single crystalline silicon membrane was released from a silicon on insulator (SOI) wafer in deionized water at room temperature. The floating membrane was then collected by another wafer (n-type doped, *N*_D_∼10^19^ cm^**−**3^) with patterned silicon bottom electrodes that was covered with chemically produced silicon oxide, followed by the patterning and etching of the membrane into top wire electrodes. The silicon membrane releasing process was of high yield (Fig. 1a) and led to large area (chip scale) single crystalline silicon membranes. The fabrication process is described in more detail in Methods, Supplementary Figs 1 and 2 and Supplementary Note 1. The cross-point device has a junction area of 5 μm × 5 μm (Fig. 1b), with a 5-nm-thick silicon oxide sandwiched between a 70-nm-thick p-type (100) silicon and n-type (111) silicon wires (Fig. 1c).

The device exhibited repeatable unipolar switching after initiating the first SET process by applying a +8 V voltage on the p-Si top electrode with 10^**−**5^ A compliance current (Fig. 1d). This unipolar switching characteristic is consistent with our previous results on devices with 1 nm silicon oxide as switching layer and Pt as top electrode^35^. A typical turn on voltage (*V*_SET_) was 7.5 V and a turn off voltage (*V*_RESET_) was 4.5 V. The programming voltage, especially the *V*_RESET_ included a large portion of voltage that was dropped on the silicon electrodes, and the actual voltage across the junction during RESET was estimated to be only 2 V. This voltage could be further reduced by using a thicker electrode and/or a thinner oxide layer. It is noteworthy that the device is considered forming-free as the first SET voltage was almost the same as the subsequent SET voltages. More interestingly, the device showed self-rectifying characteristic (Fig. 1d), that is, the reverse current under negative voltage (on the p-Si top electrode) was significantly suppressed as compared with the forward current. The rectifying ratio and the ON/OFF conductance ratio are 10^5^ and 10^4^ (both read at 2 V), respectively (Fig. 1e). The self-rectifying behaviour provides an alternative and effective approach to solve the sneak path problem without introducing external selectors, and hence enables large size crossbar arrays as confirmed by both simulation and experimental measurement of single-layer and multiple-layer crossbar arrays in the following sections. The switching behaviour was repeatable after 100 cycles (Fig. 1f) and the retention time of the device was measured at 300 °C up to 2 × 10^5^ seconds without any noticeable degradation (Fig. 1g). The device can also be programmed with electrical pulses (see Supplementary Fig. 3 and Supplementary Note 2). The typical transient resistance switching behaviour is shown in Fig. 1h for SET process (7.6 μs at 10 V) and Fig. 1i for RESET process (490 μs at 7 V). During the SET process, an external resistor was connected in series to limit the current and protect the device from hard breakdown, and the pulse amplitude was limited to 10 V by our equipment. The switch speed is heavily dependent on the pulse amplitude (exponential relationship between switching speed and pulse amplitude is expected^36^), and faster switching could be achieved by applying pulses with higher amplitudes. The relative slow switching speed and the exceptional retention performance at high temperature suggest a stable chemical composition in both states, and would be desired for applications under extreme conditions.

### Switching mechanism study

**Figure 2:**
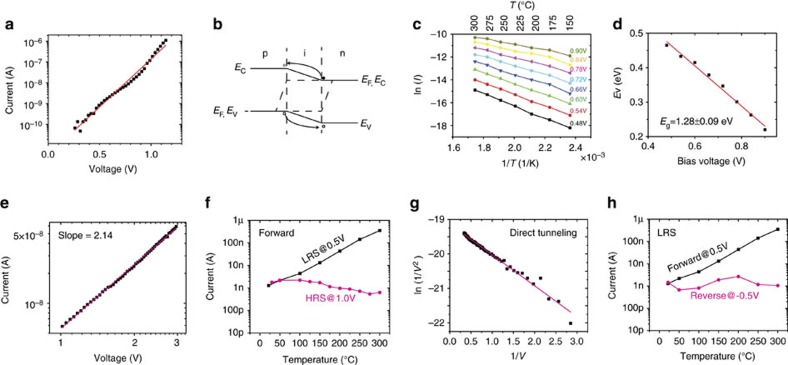
Conduction mechanism study based on IV characterization. (**a**) When the device is in LRS and under a forward bias, the current increases exponentially with voltage following the diode conduction equation. (**b**) The band diagram in LRS under a forward bias describes a piece of non-degenerate silicon bridges between two degenerate silicon electrodes. The excess electrons flow from n-type electrode to p-type electrode while the holes flow in the other direction. (**c**) The forward LRS current increases with temperature under different bias voltages. (**d**) The activation energy extracted from **c** decreases with the bias voltage. The zero-biased activation energy is about 1.28 eV and the diode ideal factor is about 1.71. (**e**) The IV relationship when the device is in HRS and under a forward bias best fits the Mott–Gurney law (space charge limited transport). (**f**) The temperature-dependent measurement when a positive voltage is applied on the TE (forward biased). The HRS current slightly decreases with temperature, consistent with the SLCL theory with few traps. The LRS current follows the diode conduction equation, which increases significantly with temperature. (**g**) The IV relation when the device is under a reverse bias best fits the Fowler–Nordheim tunnelling equation. (**h**) The temperature-dependent measurement when the device is in LRS and biased under different polarity. The reverse current fluctuates around 1 nA as temperature changes from room temperature to 300 °C, while the LRS forward current increases orders of magnitude. The weak temperature dependency under a reverse bias is consistent with the Fowler–Nordheim tunnelling.

**Figure 3:**
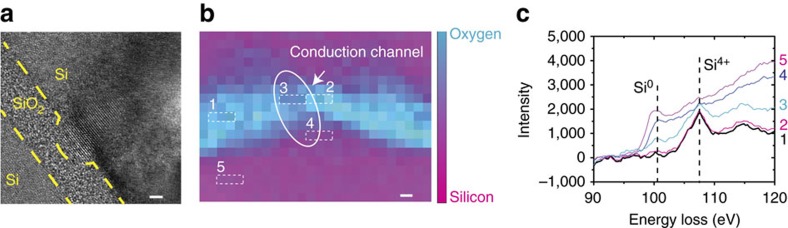
Direct observation of the silicon-rich conductive channel. (**a**) HRTEM image shows a protrusion on the top electrode side, suggests possible filamentary conductive area. The silicon is reduced from silicon dioxide under highly localized electric field and crystallizes with current generated Joule heating. (**b**) The STEM-EELS mapping for silicon and oxide elements corresponds to box area shown in the Supplementary Fig. 4a. The magenta colour indicates silicon element while turquoise indicates the oxide. The mapping clearly shows there forms a silicon protrusion on one side of the silicon electrodes. The areas 1 and 2 are typical areas for the switching material (silicon dioxide) that far from and next to the possible conduction channel, respectively. The area 3 is a typical area on the possible conduction channel. The area 4 is a representative area for the silicon protrusion, while area 5 is the bulk silicon substrate. (**c**) Spatially resolved spectra of Si L2,3 edge corresponding to the regions marked in **b**. The spectra in the channel (region 3, for example) shows significantly more Si^0^ than that in all other regions between the two electrodes (including region 1 and 2). Scale bars, 2 nm. HRTEM, high-resolution TEM.

To understand the switching mechanism of the all-silicon-based memristor, we carried out temperature-dependent IV measurements, fitted the data according to the conduction models and performed TEM studies. The forward conduction in the low-resistance state (LRS) is diode-like while that in the high-resistance state (HRS) is space-charge limited conduction (SCLC). The reverse conduction in both states is based on tunnelling. The forward IV data for low forward applied voltages shown in Fig. 2a fits well with diode equation 
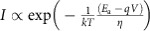
 (where *E*_a_ is the activation energy, *η* is the diode ideal factor, *q* is the elementary charge, *k* is the Boltzmann constant and *T* is temperature). Since both the top and bottom electrodes are degenerate silicon, the diode-like conduction behaviour suggests that a conducting channel made of non-degenerate semiconductor is formed in between. We believe the conducting channel is made of silicon reduced from the silicon oxide layer. An energy band diagram for this proposed diode under forward bias is illustrated in Fig. 2b. In this scenario, excess electrons flow from n-type bottom electrode (BE) to p-type top electrode (TE), while holes flow in the other direction. The temperature dependence measurement in Fig. 2c shows that the current increases with temperature, which is consistent with the diode conduction mechanism. The extracted activation energy at zero bias is 1.28±0.09 eV (Fig. 2d), close to the energy bandgap of silicon, which agrees with our theory that the conducting channel is made of silicon. The extracted series resistance is 560 Ω, consistent with the measured wire resistance between two contact pads on the same electrode. In the HRS, on the other hand, the IV relation was best fitted into a SCLC model (the Mott–Gurney law^37^ described in 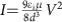, where 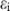 is the dielectric constant, *μ* is the electron mobility and *d* is the thickness of the dielectric), with a slope of 2.14 in the logarithmic current–voltage plot (suggesting 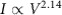) (Fig. 2e). The current is weakly dependent on temperature, as shown in Fig. 2f, which further confirms the SCLC conduction for HRS under a forward bias. Under a reverse bias, however, the current is dominated by tunnelling instead of diode reverse leakage current. The IV relation can be described in the Fowler–Nordheim tunnelling^38^ equation: 
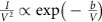
, as confirmed by the current data fitting under a reverse bias (Fig. 2g) and the weak temperature dependency (Fig. 2h). To visualize the conductive filament, we made devices with a 100 nm × 300 nm active area and used a focused ion beam (FIB) to cut out the switching area after the device was set to LRS. We have searched the whole junction area under the TEM, and have spotted only one protrusion made of crystalized silicon between the two electrodes in each device as shown in Fig. 3a. The same observation was made from devices in two different batches, suggesting the repeatability of the phenomenon. Figure 3b shows electron energy loss spectroscopic (EELS) mapping of the switching area (see Supplementary Fig. 4 for more details) obtained in a field-emission scanning TEM (STEM) operated at 200 keV. The EELS spectra (Fig. 3c) further reveals that noticeable amount of zero valence silicon exists in the remaining SiO_x_ region between the silicon protrusion and the other electrode, confirming the formation of a sub-5 nm localized Si^0^-rich condition chancel there.

On the basis of the observation, we believe that the high electric field (∼15 MV cm^−1^) during the SET process breaks some Si–O–Si bonds, pulls some oxygen atoms out of the silicon oxide matrix, and eventually results in the formation of the localized Si^0^ conduction path. This conduction path, together with the two differently doped electrodes, forms the p-i-n like diode. The current flow through highly localized filament generates significant amount of heat (a current of 10^**−**6^ A flowing through the estimated 100 nm^2^ cross-section of the conduction path leads to a current density of 1 MA cm^−2^) sufficient to melt and crystallize the silicon in an epitaxy mode^39^. The nearly round shape of the silicon protrusion (conduction channel) is a result of minimization of interfacial free energy, in agreement with the occurrence of a melting process during SET. The RESET process, on the other hand, is related to the thermal breakdown of the conductive path, especially in the region 3 (Fig. 3b) that has a weaker signal of zero valence silicon (which indicates more defects and structural weakness). The explanation is consistent with previous studies on MOS transistor breakdown^40^, which suggests that under a high electric field, oxygen could be pulled out of the silicon oxide matrix, leaving behind suboxides, defects and dielectric breakdown-induced epitaxy.

### 3D Si/SiO_2_/Si crossbar arrays

**Figure 4:**
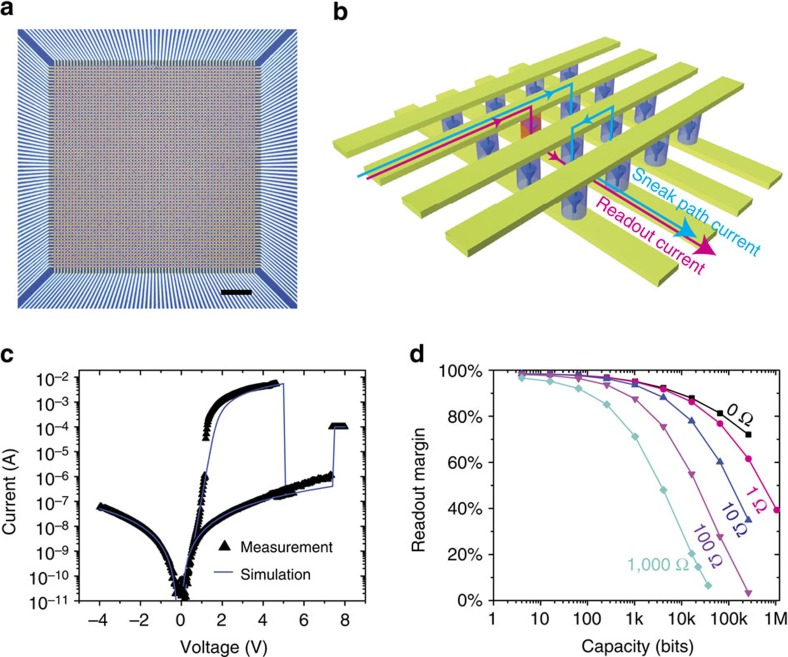
Single-layer all-silicon crossbar array and array size evaluation. (**a**) A 64 × 64 p-Si/SiO_2_/n-Si crossbar array. The devices in the arrays have a junction area of 5 μm × 5 μm. (**b**) Schematic of a crossbar array in which the sneak path problem is alleviated by intrinsic diodes at each cell. The blue line is one examplary sneak path that includes one reverse biased cell, which significantly reduces the sneak path current. (**c**) The simulated single device DC sweep IV (solid line) curve from the SPICE simulation matches the measurement data (triangles), validating the SPICE model. (**d**) The normalized readout margin is larger than 39% for a 1 Mbits crossbar array if we consider wire resistance between each cell to be 1 Ω, 27% for a 64 kbits array with 100 Ω, and 10% for 30 kbits array with 1,000 Ω. Scale bar, 100 μm.

**Figure 5:**
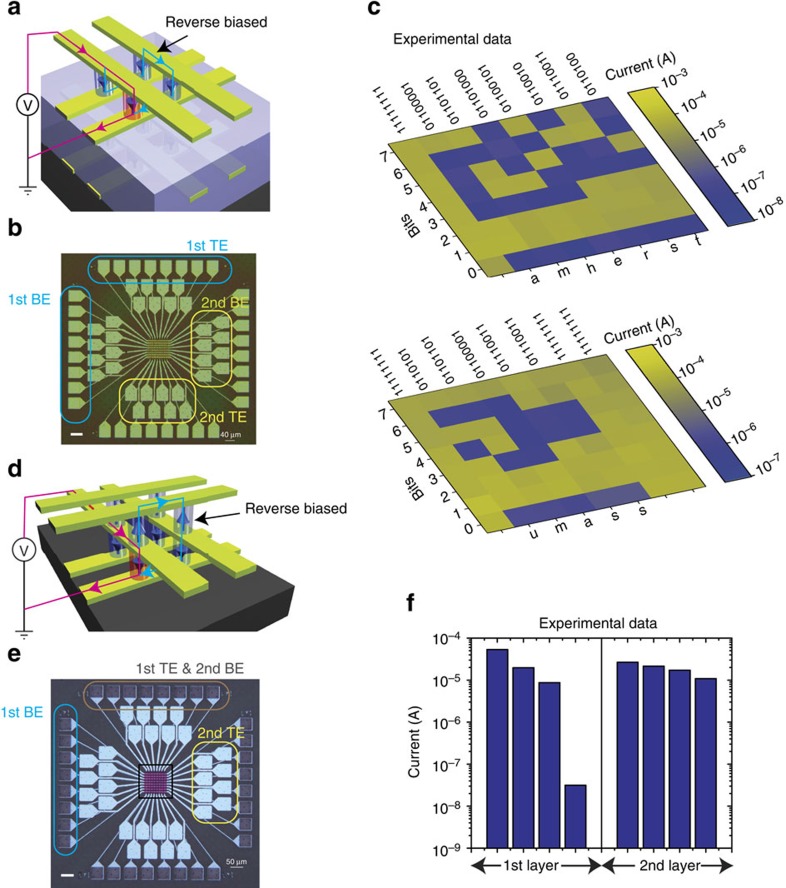
Experimental demonstration of intra- and inter-layer sneak path blocking in stacked crossbar arrays. (**a**) Schematic of the twp-layer stacked memristor crossbar array where the two layers of devices are electrically isolated by SOG. The red line is the expected current path during the readout of the selected device (red), while the blue line shows one typical intra-layer sneak path being blocked by a reverse biased cell. (**b**) Optical image of the two-layer stacked 8 × 8 memristors array with the outer contact pads connecting with the 1st layer devices while the inner pads with the second layer devices. (**c**) The colour map of the readout current by read a voltage of +2 V for the two layers stacked memristors crossbar array. Yellow represents higher read current and lower-resistance state while red the opposite. Before reading, the array was programmed into ASCII code representing ‘umass' and ‘amherst' respectively. The remaining cells were programmed into LRS to emulate the worst-case scenario with maximum sneak path current. The bits were read out correctly which proves the effective blocking of the intra-layer sneak path current by the built-in diode. (**d**) Schematic of the two-layer stacking with shared electrodes with adjacent layers. The reverse biased diode along the inter-layer sneak path (blue) prevents the inter-layer sneak path current, while the red line shows the expected path during the readout of selected device (red). (**e**) Optical image of the two-layer stacked crossbar array with shared electrodes. The connection of the contact pads was labelled in the image. (**f**) The experimental measurement result in a 2 × 2 sub-array shows that, in the worst-case scenario, the only HRS cell in the first layer can be readout correctly although all other cells are in LRS. This result confirms the successful suppression of the inter-layer sneak path current in the array. Scale bars, 50 μm. SOG, spin-on glass.

The high rectifying ratio of our device, together with the high ON/OFF conductance ratio, enables the operation of large crossbar arrays. We first evaluated the capacity of a single-layer all-silicon device crossbar array (a fabricated 64 × 64 array with high yield is shown as an example in Fig. 4a). When an individual cell in the crossbar array is accessed by applying a read/write voltage across a selected word line and bit line while all other word/bit lines are floating, current sneak paths (a possible one is illustrated with a blue line in Fig. 4b) co-exist with the selected current path (red line) through unselected devices, leading to access problems and more power consumption. For our self-rectifying unipolar device, however, the sneak path current is effectively suppressed because all the possible sneak paths inevitably include at least one reversely biased cell (diode at each junction in Fig. 4b). We built a SPICE model for the circuit simulation (described in detail in the Supplementary Fig. 5 and Supplementary Note 3) and validated the model by comparing the simulated and experimental behaviour of a single device (Fig. 4c). As shown in Fig. 4d, the readout margin is larger than 27% for a 64 kbits array if the wire resistance between two adjacent cells is 100 Ω. The readout margin is 10% for a 30 kbits array if the wire resistance is 1,000 Ω, still meeting the generally acknowledged minimum criterion of 10% to differentiate the states^27^,^41^. Our results indicate that the self-rectifying characteristic of our device effectively suppresses the sneak path current and enables larger array operation without discrete selectors. Furthermore, the single-crystal Si wires could be made sufficiently thick to reduce the wire resistance without decreasing the cell density in the array. The readout margin could also be greatly improved by using different biasing schemes (see Supplementary Fig. 6 for details). More importantly, we experimentally confirmed that both intra- and inter-layer sneak paths in multilayer Si/SiO_2_/Si memristor crossbar arrays can be effectively suppressed by the built-in diodes at each junction. We fabricated two two-layer 8 × 8 arrays by repeating the stacking fluid-supported silicon membranes and patterning processes (see Methods), one with a spin-on glass in between the two layers (array A, Fig. 5a,b and Supplementary Fig. 7a,b) and the other without (array B, Fig. 5d,e). Aluminium was deposited on the exposed contact pads and fan-out wires for better electrical contact with measurement probes. The two layers in array A were programmed into binary bits representing ASCII (American Standard Code for Information Interchange) code for ‘umass' and ‘amherst', respectively, with LRS representing logic ‘1's and HRS for ‘0's. Even though the remaining cells were programmed into LRS on purpose (to emulate the worst-case scenario with maximum sneak path current), the programmed information was correctly read out by applying a 2 V read voltage across the selected top and bottom electrode pads, while leaving all others floating. The successful readout (Fig. 5c and Supplementary Fig. 7c) of all the HRS cells proves that the built-in diodes within the memristor cells effectively blocked the intra-layer sneak path current. For array B, only three layers of silicon electrodes with n-p-n types of doping were used to construct two crossbar layers, with the middle acting as both the top electrode of the first-layer device and the bottom electrode of the second. Because of the different doping types of the neighbouring electrodes, at least one of the built-in diodes (for example, circled cell in Fig. 5d) is reversely biased along the inter-layer sneak path and hence blocking its sneak current. We programmed a 2 × 2 sub-array in array B with one device in the first layer into HRS and all seven other into LRS, to emulate a worst-case scenario. The device in HRS was successfully read out (Fig. 5f), confirming the effective blocking of inter-layer sneak paths by the built-in diodes.

**Figure 6:**
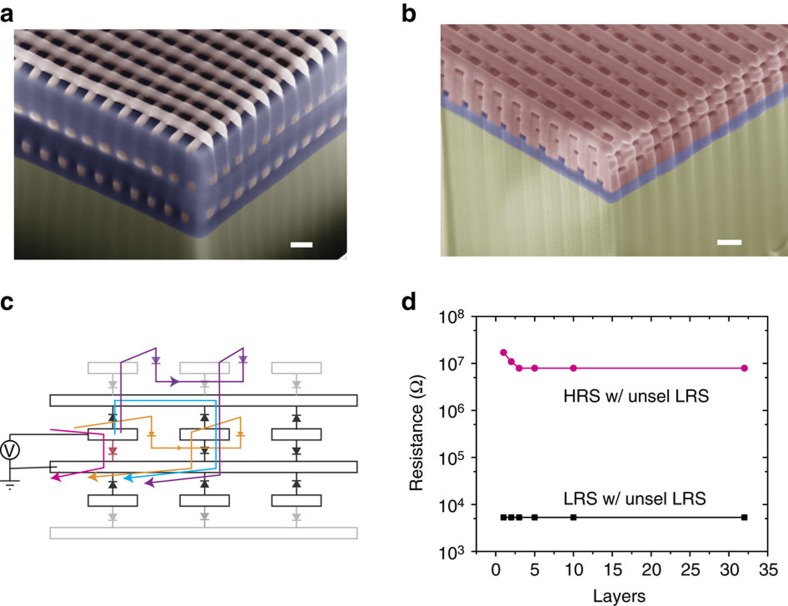
Demonstration of multiple-layer Si/SiO_2_/Si nanoscale crossbar arrays. (**a**) The fabrication results for the 3D stacked silicon crossbar devices with isolation between different layers. The Si nanowires is 100 nm wide and 70 nm thick with a pitch of 200 nm. (**b**) The fabrication results for the 3D stacked silicon crossbar devices with shared electrodes with adjacent layers. The geometry of the nanowires is the same as that in **a**. (**c**) Schematic representation for 3D stacking with shared electrodes between adjacent layers. The blue curve shows one possible inter-layer sneak path and the orange one the intra-layer sneak path. The purple line shows a sneak path go through beyond one layer on top of selected layer will inevitably include at least 3 reverse biased device, which limits the sneak path current. (**d**) Simulated readout resistance in different states in a 64 × 64 array with different layer number considering the worst-case scenario. The readout result shows little difference when the layer number is larger than 3. Scale bars, 200 nm.

Finally, we built five layers of crossbar arrays with nanoscale Si/SiO_2_/Si memristors, demonstrating the potential of stacking nanodevices into 3D over large areas using the fluid-supported membrane transfer technique. Figure 6a shows a cross-sectional SEM image for a 3D stacked silicon crossbar arrays with a layer of hydrogen silsesquioxane (HSQ) in between each crossbar layer. The HSQ was used to electrically isolate different layers, and to planarize the sample before fabricating the next layer on top. With such isolation, the six layers of silicon nanowires made a total of three crossbar layers built on the same footage, greatly increasing the packing density of the devices. To further increase the packing density and to simplify the fabrication process, we eliminated the HSQ layer and stacked the electrodes with alternating p- and n-type silicon nanowires (Fig. 6b). Mechanically, the fabrication of the 3D array was enabled by the high Young's modulus of the single crystalline silicon nanowires that prevents the 100 nm wire from bending down when arranged into a 200 nm pitch (see Supplementary Fig. 8 for a cross-sectional TEM images). Electrically, alternating of the p- and n-type silicon wires makes it possible to share a middle layer of silicon wires by two adjacent crossbar layers without inter-layer sneak paths. As such, the six layers of silicon nanowires forms five layers of crossbar devices, further enhancing the packing density. Our extensive simulation for a 64-by-64 array with all possible sneak paths (Fig. 6c shows typical ones) taken into account shows that the readout margin for 3D crossbar array with alternating p- and n-Si electrodes remain the same when the layer number is larger than 3 (Fig. 6d). Our 3D stacking results demonstrate not only the stackability of the 3D array using a simplified fabrication process, but also the feasibility of electrical operation of such multilayer arrays without introducing external selectors.

The Si/SiO_2_/Si memristors offer full CMOS compatibility and more importantly, repeatable self-rectifying unipolar resistive switching with a large memory window. These features enable massively parallel crossbar arrays that can be used for memory and computing applications without external selector devices. Compared with other recently reported self-rectifying devices, our p-Si/SiO_2_/n-Si device excels at a number of figure of merits such as ON/OFF ratio, rectification ratio and retention (Supplementary Table 1). The built-in diode at each junction eliminates the sneak paths during the write operation and hence negates the necessity of using external selector devices, bringing tremendous benefit in the circuit design and fabrication simplicity. There have been a few reports that used polysilicon to build self-rectifying devices^42^, but the approaches were either not 3D stackable, or involved high temperature that is incompatible with BEOL CMOS process. On the basis of the fluid-supported single-crystalline silicon membrane transfer technique, our approach is at room temperature, fully CMOS compatible and 3D stackable. The fabrication method is applicable to other substrates such as plastics because of these advantages. The 3D arrays, without intra- and inter-layer sneak paths, offer significantly increased packing density for the devices in both memory and computing applications.

## Discussion

In summary, we have demonstrated Si/SiO_2_/Si memristor device and arrays that are fully CMOS compatible in both materials and fabrication procedure. We systematically studied the switching mechanism and confirmed that the switching is due to the formation and rupture of a sub-5 nm silicon-rich conduction channel inside of the oxide layer. We experimentally demonstrated that the devices can be stacked into operational 3D crossbar arrays, and the self-rectifying switching behaviour alleviates the sneak path problem without discrete selector devices. The avoidance of selectors will greatly increase the device packing density, reduce circuit complexity, and lower down the manufacturing cost. The device fabrication and integration is ready for CMOS foundry on full-wafer scale silicon wafers. We also believe that the technology is applicable to a broader variety of substrates and materials, opening up opportunities in 3D flexible and wearable electronics.

## Methods

### Device fabrication

The n-type Phosphorous doped <111> silicon substrate (*N*_D_∼10^19^ cm^**−**3^) was used as bottom electrodes. A 150-nm-thick silicon oxide was deposited by PECVD (parameter: 2% SiH_4_/N_2_: 400 sccm, N_2_O: 1,420 s.c.c.m., power: 30 W, pressure: 800 mtorr) as an isolation layer and then was patterned by photolithography and reactive ion etching (RIE) to define the window to make the junction. A Piranha cleaning (three part H_2_SO_4_ and one part H_2_O_2_ in volume ratio) and a short dip in 1:50 HF were conducted to clean the wafer. The silicon oxide switching layer was then produced from the N-type silicon wafer in Piranha solution for 20 min, followed by an oxygen plasma treatment (O_2_: 50 s.c.c.m., power: 40 W, pressure: 4 mtorr) for 5 min.

The top electrode was transferred from another SOI wafer (commercial Soitech smart-cut wafer with a 70-nm-thick device layer and a 140-nm-thick buried oxide (BOX) layer). The SOI wafer was doped by Boron ion implantation (dose: 2 × 10^15^ cm^**−**2^, energy: 10 keV, tilt: 7°) followed by a rapid thermal processing (1,100 °C, 30 s). The resulting *N*_A_ was about 10^20^ cm^**−**3^. The SOI wafer was then patterned into mesh structure (3 μm diameter holes with 25 μm pitch and 75 nm depth, to facilitate membrane releasing) by photolithography and RIE, cleaned with Piranha solution, and immersed into concentrated hydrofluoric acid solution (49% HF: DI water=1:1) for 40 min. After that, the wafer was transferred into deionized (DI) water, and the silicon device layer separated from the bulk automatically and floated on the surface of DI water due to surface tension of the water. The floating silicon membrane was transferred to the N-type silicon wafer with bottom electrodes using tweezers, and dried using a nitrogen blow gun. The sample was then baked at 110 °C for 5 min, cleaned by ultrasonic in acetone, and patterned by photolithography (aligned with BE patterns) and RIE. Because the thin layer of silicon (70 nm thick for our case) is optical transparent, we were able to do the alignment with the bottom electrodes pattern originally on the substrate. Finally, Al contact pads (100 nm thick) were fabricated on the contract pads by photolithography, evaporation and lift-off.

### Electrical characterization

A Keithley 4200SCS and an Agilent 4156B semiconductor parameter analysers were used. The measurements were performed on a probe station under ambient condition unless otherwise specified. The IV characteristic measurement of the p-Si/SiO_2_/n-Si was carried out by applying a bias voltage (sweep or pulse) on the top electrode (p-Si) while bottom electrode (n-Si) was grounded. For temperature dependence and retention measurements, an Agilent B1500 with a Cascade summit probe station with a heated sample stage (ambient to 300 °C, 0.1 °C accuracy) was employed.

### SPICE modelling

The device model was described with Verilog-A., The conduction and switching model and corresponding parameters were chosen by comparing the electrical measurement data with difference models. Synopsis HSPICE was used in circuit simulation, and the netlist file was generated by self-written Python script. More details are provided in the Supplementary Note 3.

### Physical characterization

The TEM samples were prepared with FEI Helios 600 NanoLab. We made 100 nm × 300 nm devices for TEM characterization, so that the possible conductive filament will be more likely confined within the ∼50-nm-thick FIB cut sample. The device was SET to LRS, protected with a layer of Pt (by electron beam assisted deposition followed by ion beam assisted deposition), and cut with gallum ion beam (30 keV). The slices were then thinned to about 30 nm under 3 keV to minimize the gallium contamination. The high-resolution TEM image and spatially resolved EELS spectra were acquired in a JEOL JEM-2100 F operated at 200 keV.

### 3D stacking of silicon crossbar arrays

The bottom electrodes of the first crossbar layer were patterned on an SOI wafer of n-type doped device layer with electron beam lithography (EBL) and RIE. Cross-alignment marks were also designed and patterned on this layer. The p-type doped silicon membrane was then transferred to this wafer using the aforementioned method, which was patterned with EBL into top electrodes of the first crossbar layer, with designed cross-alignment marks aligned to those on the lower layer. The 70 nm thick silicon membrane is transparent to the 30 keV electron beam, so the alignment marks are visible under SEM. The following layer was then transferred and patterned using the same procedure except for alternating the doping type of the transferred silicon membrane. For some crossbar arrays, a∼340-nm-thick HSQ layer was spun on and baked at 400 °C as the isolation and planarization layer after transfer and patterning of the top electrodes in each layer. The HSQ in the alignment mark area was selectively etched away with HF in order to make the alignment visible during the following EBL process.

### Data availability

The data that support the findings of this study are available from the corresponding author upon request.

## Additional information

**How to cite this article:** Li, C. *et al*. Three-dimensional crossbar arrays of self-rectifying Si/SiO_2_/Si memristors. *Nat. Commun.* **8**, 15666 doi: 10.1038/ncomms15666 (2017).

**Publisher's note**: Springer Nature remains neutral with regard to jurisdictional claims in published maps and institutional affiliations.

## Supplementary Material

Supplementary Information Supplementary Figures, Supplementary Table, Supplementary Notes and Supplementary References
