# Supplementary material for: Three-dimensional crossbar arrays of self-rectifying Si/SiO2/Si memristors
Source: Nat Commun. 2017 Jun 5;8:15666. doi: 10.1038/ncomms15666 (PMC5465358; doi:10.1038/ncomms15666)
Supplement: Supplementary Information — Supplementary Figures, Supplementary Table, Supplementary Notes and Supplementary References [file ncomms15666-s1.pdf]

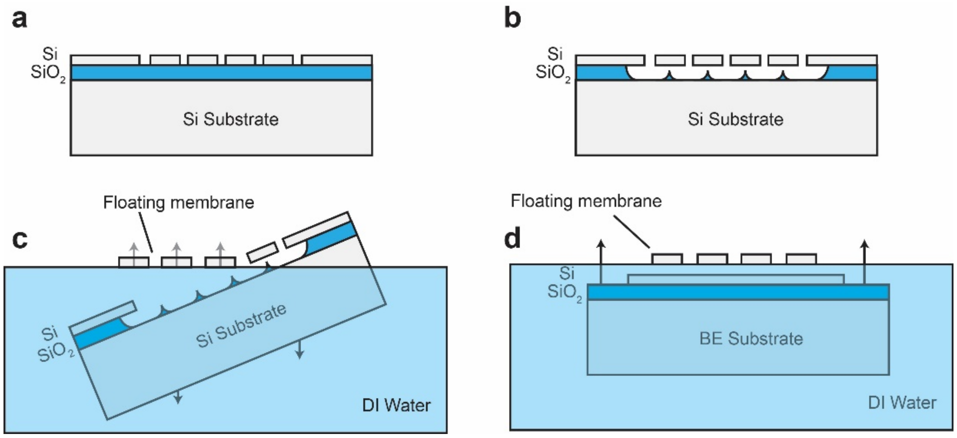

Supplementary Figure 1 | The schematic of membrane release and transfer processes. **a**, An SOI wafer is patterned into mesh structure using photolithography and dry etching. **b**, HF etching undercuts buried oxide through the patterned holes. **c**, The silicon membrane is peeled off from the substrate by capillary force, and floats on the surface of the DI water; **d**, The membrane is picked up by another substrate.

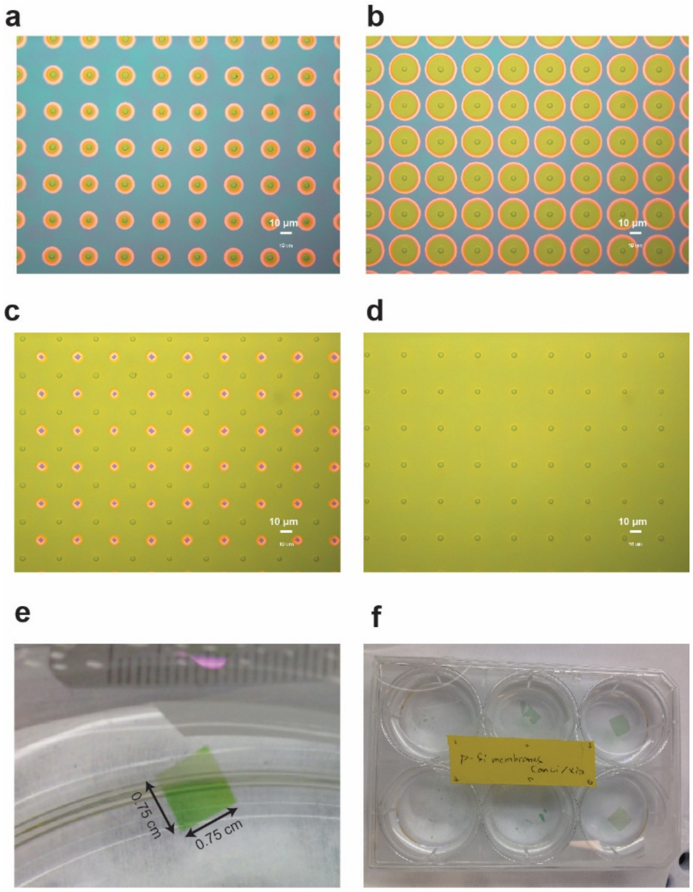

Supplementary Figure 2 | Silicon membranes. a-d, Optical microscope images show that the silicon oxide under the silicon device layer of SOI wafer was gradually etched away, and the silicon membrane was finally fully released. **e**, A freestanding 0.75 cm×0.75 cm single-crystalline silicon membrane floated on DI water in a beaker. **f**, The membranes stored in a DI water container for future use. Scale bars, 10 μm.

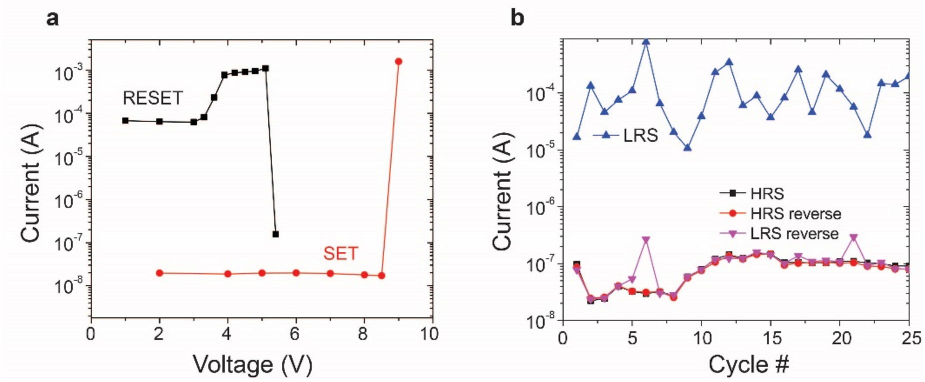

Supplementary Figure 3 | Resistive switching by electric pulses for a 5×5 μm^2 device. a, Switching behavior when 5 ms electric pulses were applied on the top (p-type) silicon electrode. The current was read by applying a 2 V pulse on the top electrode. The RESET and SET voltages were determined to be 5.5 and 9.0 V, respectively. **b.** 25 consecutive cycles of the pulse switching. The current was read under +2V (forward) and -2V (reverse) voltage pulses on the top electrode after each write operation. The reverse current was suppressed regardless of the resistance state of the device.

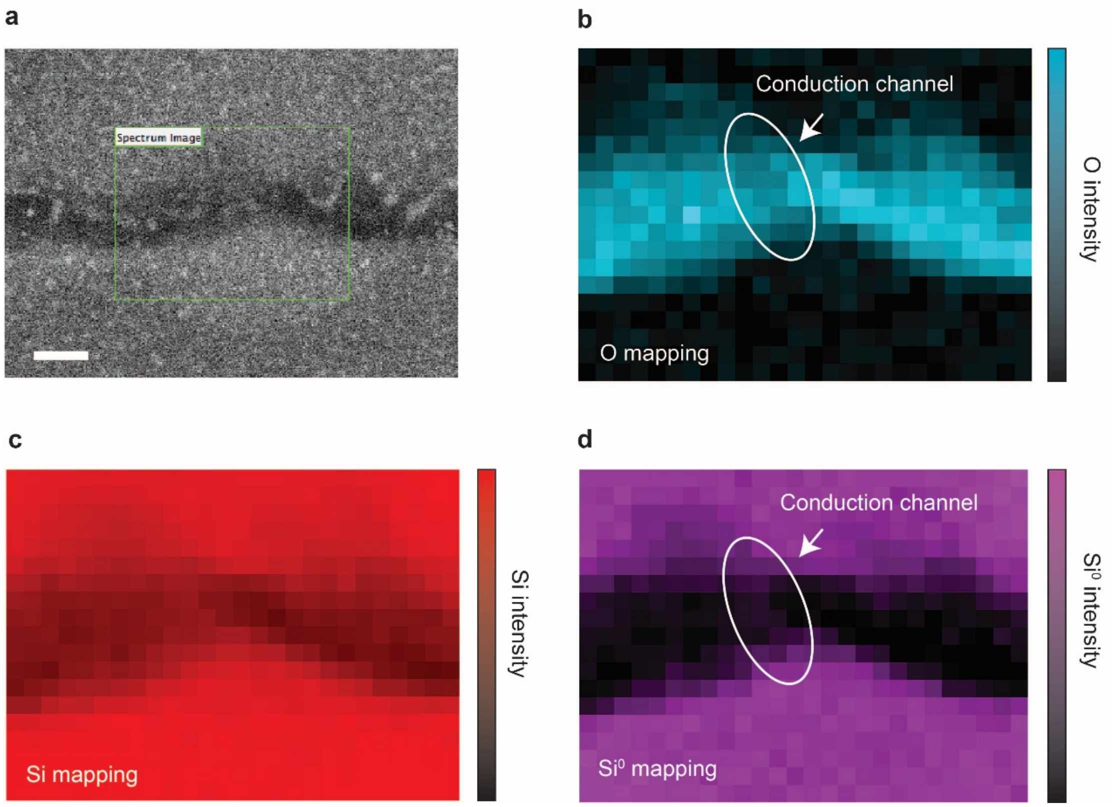

Supplementary Figure 4 | Additional STEM images and EELS elementary mapping. a, The annular dark-field scanning TEM (STEM) image of the switching location. The square shows the corresponding STEM-EELS mapping area. **b**, The 2D EELS mapping for oxygen element shows a protrusion area with almost no oxygen and a channel connecting two electrodes with less oxygen, which we believe is the conduction channel. **c**, The EELS mapping for silicon element shows the protrusion found in the oxygen mapping is made of silicon. However, the channel found in oxygen mapping is not clear. **d**. The EELS mapping for zero valence silicon shows noticeable zero valence silicon constructing the channel, confirming the channel is Si^0 rich. (Spatially resolved spectra of Si L2,3 edge are presented in Figure 3 in the main text.). Scale bar, 10 nm.

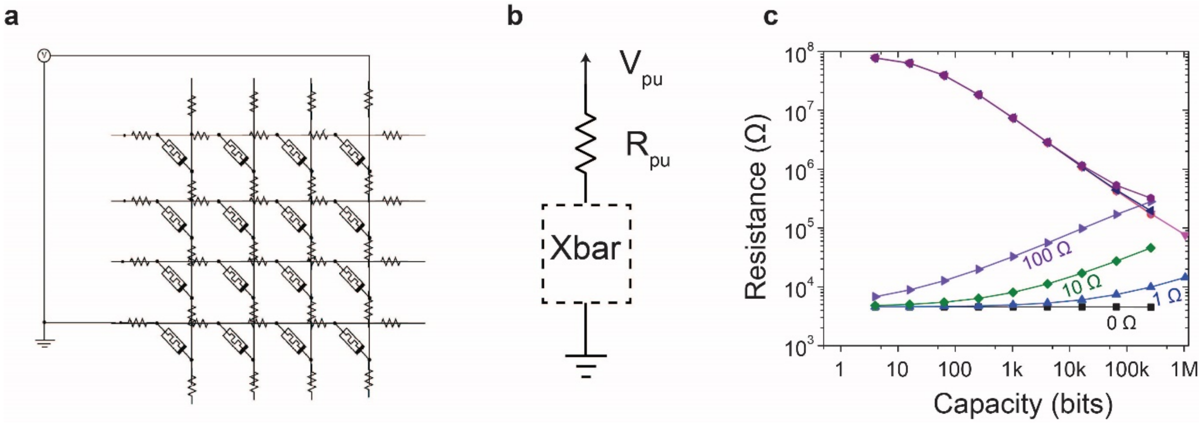

Supplementary Figure 5 | Crossbar array size estimation. **a**, The circuit used in the SPICE simulation for the array size estimation. The wire resistance between each memristor node is between 0 Ω and 1000 Ω in the following simulation. The voltage is applied on the far corner device, so that there will be the most significant impact from wire resistance. **b**, The circuit schematic used during the calculation of readout margin, where a pull-up resistor is connected in series with the crossbar array, and the states of the accessed device was read by sensing the voltage drop on the pull-up resistor. **c**, The readout resistance of the selected device in a crossbar array with different size capacity and wire resistance. The HRS resistance decreases with array size due to larger sneak path current, while the LRS resistance increases with the wire resistance, because the wire resistance becomes more significant than cell resistance itself.

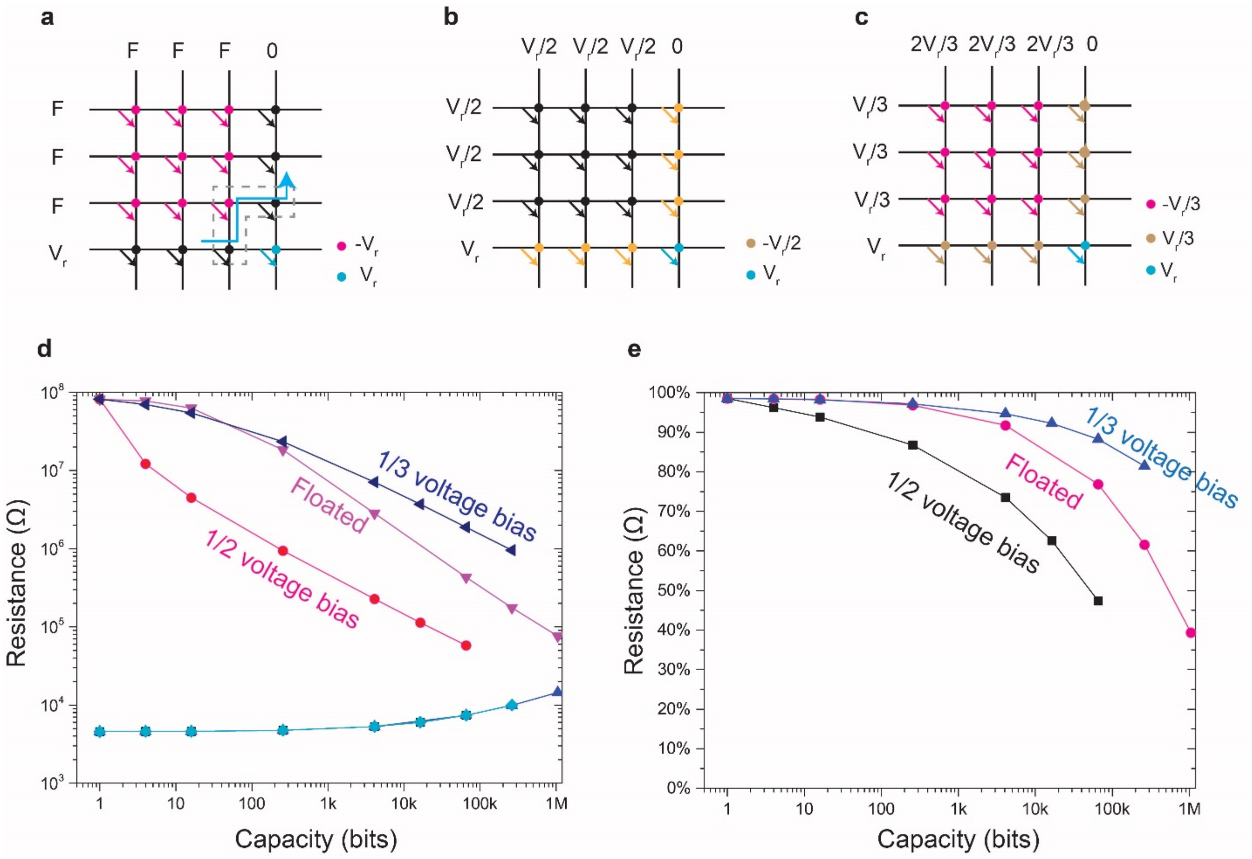

Supplementary Figure 6 | SPICE simulation for the array size estimation with different read out schemes. a, The floating scheme. The voltage is applied across the selected word and bit line while all other word and bit lines are floated. The blue staircase line shows one possible sneak path in this scenario which includes one reverse biased cell and two forward bias cell. The cells in red color are reverse biased, which suppress the sneak path current. **b**, The half voltage bias scheme. In this case, the voltage is applied across the selected word and bit line while all other bit and word lines are half voltage biased. In this case, the cells in yellow color are half biased. The half biased cells suppress the sneak path current, because of its nonlinear forward IV relation. **c,** The one-third voltage bias scheme. In this case, the selected word line is fully biased while the selected bit line grounded. The unselected word lines and bit lines are biased at 1/3 and 2/3 of the full bias voltage respectively. **d,** The readout resistance of the selected device in a crossbar array shows that the readout ON/OFF resistance ratio is larger if the 1/3 voltage bias scheme is used. **e**, The normalized readout margin with different size capacity and read scheme, which shows the readout margin can be improved by using the one-third bias scheme. In the simulation, the wire resistance between each cell node of 1 Ω is assumed.

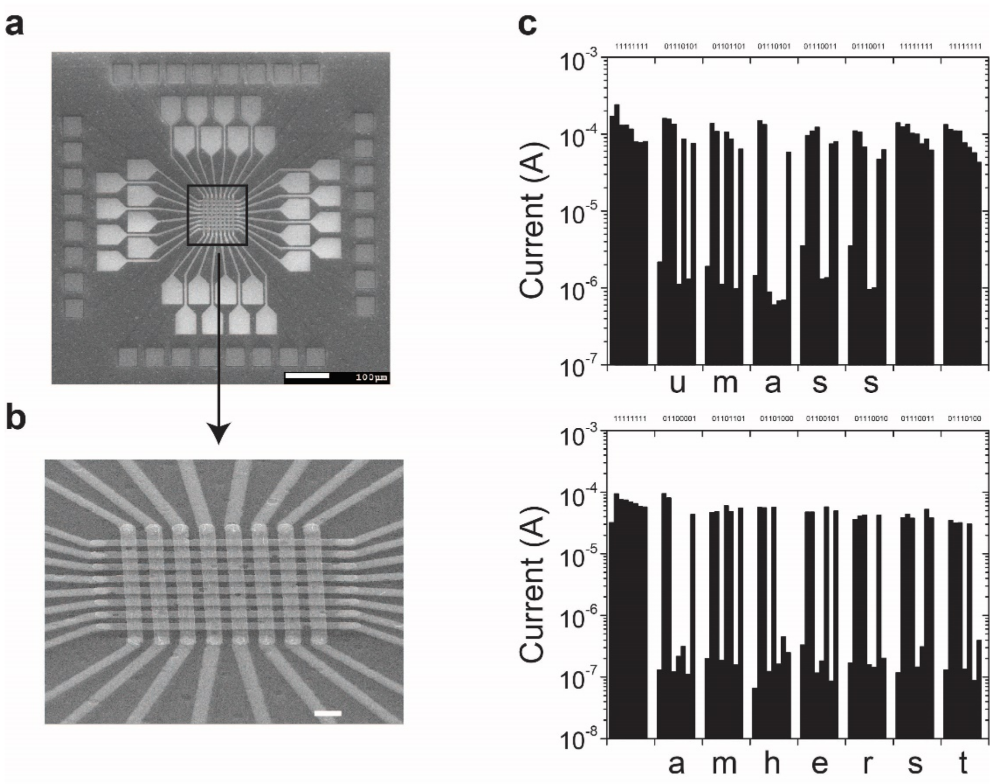

Supplementary Figure 7 | Two layers of stacked p-Si/SiO_2/n-Si crossbar memristors array. **a**, SEM image of the 2-layer stacked 8×8 memristors array. For the 1st layer (bottom layer) structure, only the measurement pads can be clearly seen in this image as other parts are buried inside of the interlayer dielectric. Scale bar, 100 µm. **b**. The zoom-in area of the SEM image for the crossbar devices. **c**. The readout current at +2V for the 2 layers stacked memristors which were programmed into "umass" and "amherst" respectively. All other cells were programmed into LRS to emulate the worst-case scenario with maximum sneak path current. Scale bar, 10 µm.

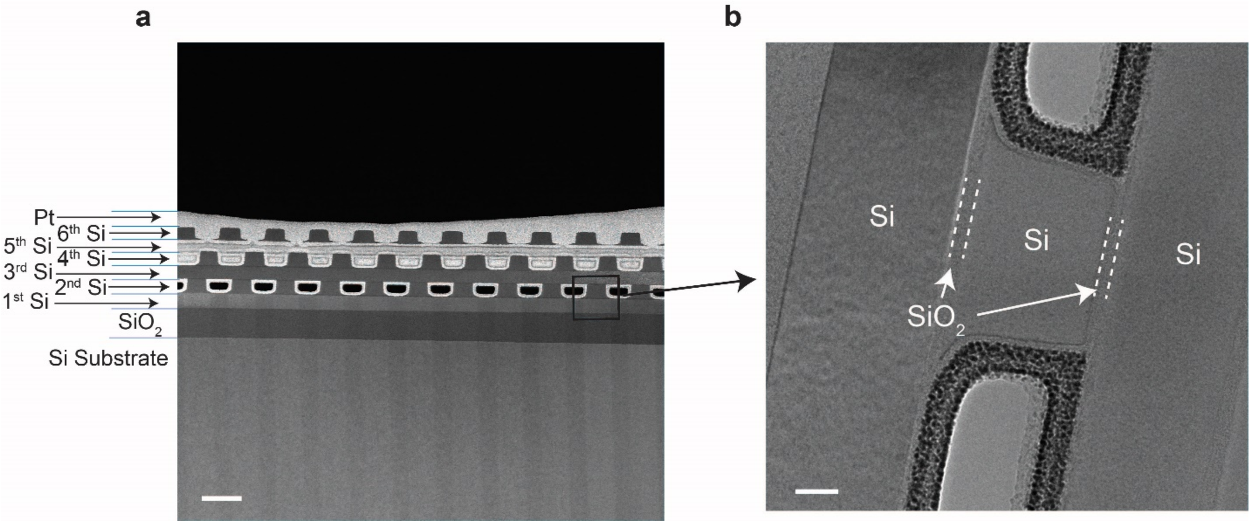

Supplementary Figure 8 | Cross-sectional TEM image of the 3D stacked Si/SiO_2/Si memristor crossbar arrays. **a**, The annular dark-field (ADF) STEM image of the cross-sectional TEM image of the 6 layers of the stacked Si that made 5 layers Si/SiO_2/Si memristor crossbar arrays. The top protective Pt layer and the bright rings in the lower layer was deposited during the TEM sample preparation with FIB using a gas injection system. The brighter color of Pt material in the ADF-STEM image indicates more conducting property, and the 5th Si layer is blocked by the Pt deposited during FIB in this image because of a slight misalignment to lower layers. The voids closer to the surface were filled up by Pt because the gas is coming from the surface area, and the holy 3D structure is permeable to the gas precursor. Scale bar, 200 nm. **b**, HRTEM image of the first 2 layers of the 3D Si/SiO_2/Si cross bar, corresponding to the area marked in the square area in **a**. About 5 nm amorphous SiO_2 is sandwiched between the adjacent Si layers. The dark dotted rings in the voids in the HRTEM image are the Pt, which is corresponding to the bright rings in the ADF-STEM image in **a**. The darker color indicates higher atomic ratio in the HRTEM image. Scale bar, 20 nm.

Supplementary Table 1 | Comparison of the performance of self-rectifying memristors in recent reports

Structure	ON/OFF ratio	Rectification ratio	Endurance	Retention	Polarity	Ref
TiW/$Ge_2Sb_2Te_5$/W			>10^3	5000 s		[1]
Pt/ZrO2/n^+-Si	10^6@1V	>10^4	N/A	10^5 s	WORM	[2]
Au/ZrO2:Au-nanocrystals/n^+-Si		700@±0.5V	100	N/A	bipolar	[3]
Si/a-Si core/Ag nanowires	10^4	10^6@±1V	10^4	>2 weeks	bipolar	[4]
Ag/a-Si/p-Si	10^3		10^6	>3 months	bipolar	[5]
Ag nanowires/a-Si/p-poly-Si	>10^6@0.5V	>10^6@±0.5V	>10^8	10^3 s @140 °C	bipolar	[6]
Ti/Si_3N_4/SiO_2/n-Si	10^2@1V	>10^2@±1V	10^3	> 3×10^4 s @ 85 °C	bipolar	[7]
Pt/TaO_x/n-Si	10^4@0.8V	>10^2@0.5V	100 (DC)	2000 s	bipolar	[8]
Cu/SiO_x/Si	>500@5V	>200@±0.5V	N/A	N/A	unipolar	[9]
Ni/HfO_2/n^+-Si	10^4@0.1 V	>10^3@1V	100 (DC)	> 2×10^4 s @ 125 °C	unipolar	[10, 11]
Cu/HfO_2/n-Si	10^4@1V	10^4@1V	>50 (DC)	> 2000 s @ 85 °C	unipolar	[12]
**p-Si/SiO_2/n-Si**	**>10^4@1.5V**	**10^5@±1.5V**	**>100 (DC)**	**> 2×10^5 s @ 300 °C**	**unipolar**	**This work**

Supplementary Note 1: Release and transfer of floating silicon membranes

Supplementary Fig. 1 is the schematic illustration of the fluid-supported membrane transfer procedure we used for our device fabrication. First, the silicon device layer on one SOI wafer was pattern into mesh structure to expose the buried oxide (BOX), which was then etched with concentrated hydrofluoric (HF) acid (**Supplementary Figs. 1a, 1b**). After the BOX layer was fully removed the SOI wafer was immersed into deionized (DI) water in another beaker. Since the silicon membrane was hydrophobic after HF etching, it was thus peeled off from the SOI wafer by capillary force in the DI water (**Supplementary Fig. 1c**). The floating membrane was then transferred to another substrate by picking it up from the beaker (**Supplementary Fig. 1d**). Moisture was removed by nitrogen blow dry and baking on a hotplate at 110 °C for 5 min. After baking, the collected membrane cannot be separated from the substrate by DI water washing or ultrasonic treatment in acetone, which suggests a strong bonding formed between the two contacted silicon surfaces. The top electrode made of transferred membrane was then patterned by photolithography and dry etch.

It is noteworthy that we can achieve high fabrication yield in the large-area silicon membranes transfer. **Supplementary Figs. 2a-2d** are optical microscope images for a patterned SOI wafer during the releasing process. The BOX was gradually removed as the etching progressed. **Supplementary Fig. 2e shows** a square silicon membrane with dimension of 0.75 cm×0.75 cm floating on the DI water in a beaker. Larger membranes are possible with a larger pre-patterned area on the SOI wafer. The silicon membrane can also be stored in a water filled box (shown in **Supplementary Fig. 2f**) for future use.

Supplementary Note 2: Additional electrical measurements

Our p-Si/SiO_2/n-Si memristive device can also be repeatedly programmed with electrical pulses. Voltage pules were applied to the devices and the currents were measured simultaneously to monitor when the devices were switched. For example, a 5×5 μm^2 device was RESET to HRS with a 5 ms/5.5 V pulse and SET to LRS with a 5 ms/9 V pulse (**Supplementary Fig. 3a**) applied on the top electrode (p-Si). The resistance states were read with 5 ms/2 V pulses that cannot change the device state. The RESET and SET voltages are higher than those measured with DC sweeps (V_{RESET}=4.5 V, V_{SET}=7.5 V at DC). **Supplementary Fig. 3b** shows 25 consecutive cycles of the pulse switching, with current read under 5ms/+2V (forward) and 5ms/-2V (reverse) voltage pulses on the top electrode after each write operation. It is clear that the reverse current was suppressed regardless of the resistance state of the device.

Supplementary Note 3: SPICE modelling and simulation for array size estimation

The SPICE model of our all silicon resistive switching device is written in Verilog-A language. The following code snippet describes the voltage-current relation in each state:

```
if (state == 0)          //HRS
begin
        if (Vtb >0)
                Itb = 5.56e-9*pow(Vtb,2.14);
        else
                Itb = -pow(Vtb,2)*exp(0.80623/Vtb-19.36638);
end
else if (state== 1)      //LRS
begin
        if (Vtb >0.001)
                Itb = 3.45e-12*exp(10.61*Vtb);
        else if (Vtb > 0)
                Itb = 3.4868E-9* Vtb;
        else
                Itb = -pow(Vtb,2)*exp(0.80623/Vtb-19.36638);
end
else                     //Breakdown
begin
        if (Vtb >0)
                Itb = Vtb/50;
        else
```

```
                Itb = -pow(Vtb,2)*exp(0.80623/Vtb-19.36638);
end
```

And this code snippet describes how the state changes with applied voltage and/or current:

```
if (Vtb > 7.3)   //set
        state =1;

if (Itb >1e-2)   //breakdown
begin
        state = -1;
        $strobe("break down, current=%f", Itb);
end
else if (Itb > 5.4e-3 ) //reset
        state = 0;
```

A resistor of 550 Ω is also added in series with the above described modelling device in order to reflect the measured series resistance within the device cell. The SPICE model well fits the measurement data, as is shown in **Fig. 4c** in the main text.

We first use floating scheme to read the resistance state of the selected cell in a crossbar array during the simulation. In this scheme, the cell state is read by applying voltage across the selected word and bit lines with all other lines floated. An illustrative 4×4 crossbar array that demonstrates the simulation algorithm is shown in **Supplementary Fig. 5a.** For all the readout simulation, the worst-case scenario is considered as follows: 1. The voltage is applied on the far most cornered device, so that the current goes through the largest wire resistance. 2. All the unselected devices are in LRS, so that the sneak path current is the largest among all the cases. A self-written python script is used to generate the netlist automatically for a large device array.

In the circuit operation, the resistance states are usually read by detecting the voltage across the pullout resistor (**Supplementary Fig. 5b**). The resistance of the pullout resistor is chosen to maximize the voltage change amplitude $\Delta V / V_{\mathrm{pu}}$, aka. readout margin. We choose the pullout resistance to be $R_{\mathrm{pu}} = \sqrt{R_{\mathrm{LRS}} \cdot R_{\mathrm{HRS}}}$ based on the following calculation in the simulation:

The readout margin:

$$\frac{\Delta V}{V_{\text{pu}}} = \left(\frac{R_{\text{pu}}}{R_{\text{pu}} + R_{\text{LRS}}} - \frac{R_{\text{pu}}}{R_{\text{pu}} + R_{\text{HRS}}} \right)$$

$$= \left(\frac{1}{1 + R_{\text{HRS}} / \eta R_{\text{pu}}} - \frac{1}{1 + R_{\text{HRS}} / R_{\text{pu}}} \right)$$

$$= \left(\frac{1}{1 + x / \eta} - \frac{1}{1 + x} \right)$$

Where η is the $R_{\text{HRS}} / R_{\text{LRS}}$ ON/OFF ratio, x is $R_{\text{HRS}} / R_{\text{pu}}$

R_{pu} should be chosen to maximize the readout margin, so

$$\frac{\partial}{\partial x} \frac{\Delta V}{V_{\text{pu}}} = \frac{1}{(1 + x)^2} - \frac{1}{\eta (1 + x / \eta)^2} = 0$$

The solution is $x = \sqrt{\eta}, \eta \neq 1$

So pull up resistance was chosen to be $R_{\text{pu}} = R_{\text{OFF}} / \sqrt{\eta} = \sqrt{R_{\text{LRS}} \cdot R_{\text{HRS}}}$

The readout resistance considering different wire resistance between each cells is plotted in **Supplementary Fig. 5c**. For the heavily doped Si (10^{20} cm^{-3} boron doping, 0.001 Ω·cm resistivity) the sheet resistance for a 70 nm thick silicon wire is around 150 Ω/sq, and in the simulation we considered the wire resistance from 0 to 1000 Ω/sq. Larger readout resistance difference in different state will yield larger readout margin by sensing the voltage drop on the pull up resistor. The readout margin is shown in **Fig. 4** in the maintext.

The readout margin can be further improved if we use different voltage bias schemes, although they may consume more power and involve more complicated circuit implementation than the floating one[13]. Here we show the comparison between floating (**Supplementary Fig. 6a**), half voltage bias (**Supplementary Fig. 6b**) and one-third voltage bias schemes (**Supplementary Fig. 6c**). The readout resistance for the selected cell in an array and the normalized readout margin for different read scheme in a different size of array are shown in

Supplementary Figs. 6d, 6e respectively. In the floating scheme, the cells in red color are reverse biased which suppress the sneak path current. The sneak path current increases with N^2, where N is the number of word and/or bit lines. In the half voltage scheme, the nonlinear forward conduction of our device cells in yellow color (half voltage biased) helped reduce the sneak path problem, and the current increase with N, which is slower than floated scheme, as is also suggested in the simulation result shown in **Supplementary Figs. 6d, 6e**. However, the performance is worse than floating scheme because of the poorer forward nonlinearity than rectifying ratio. For the one-third voltage bias scheme, both rectifying and forward nonlinearity helped reduce the sneak path current, and the simulation result suggests it is the most optimized scheme for the readout margin performance. However, it consumes the most power[13] and is also the most complicated to be implemented in circuits. A trade-off between the readout margin, power consumption, and the circuit complexity should be considered for the device using in an array.

Supplementary References

1. Yi-Chou C, *et al.* An access-transistor-free (0T/1R) non-volatile resistance random access memory (RRAM) using a novel threshold switching, self-rectifying chalcogenide device. In: *Electron Devices Meeting, IEDM '03 Technical Digest.* (2003).
2. Zuo Q, *et al.* ZrO_2-Based Memory Cell With a Self-Rectifying Effect for Crossbar WORM Memory Application. *IEEE Electron Device Lett.* **31**, 344-346 (2010).
3. Zuo Q, *et al.* Self-rectifying effect in gold nanocrystal-embedded zirconium oxide resistive memory. *J. Appl. Phys.* **106**, 073724 (2009).
4. Dong Y, Yu G, McAlpine MC, Lu W, Lieber CM. Si/a-Si Core/Shell Nanowires as Nonvolatile Crossbar Switches. *Nano Lett.* **8**, 386-391 (2008).
5. Jo SH, Lu W. CMOS Compatible Nanoscale Nonvolatile Resistance Switching Memory. *Nano Lett.* **8**, 392-397 (2008).

6. Kim K-H, Hyun Jo S, Gaba S, Lu W. Nanoscale resistive memory with intrinsic diode characteristics and long endurance. *Appl. Phys. Lett.* **96**, 053106 (2010).

7. Kim H-D, Yun M, Kim S. Self-rectifying resistive switching behavior observed in Si3N4-based resistive random access memory devices. *J. Alloys Compd.* **651**, 340-343 (2015).

8. Gao S, *et al.* Forming-free and self-rectifying resistive switching of the simple Pt/TaO_x/n-Si structure for access device-free high-density memory application. *Nanoscale* **7**, 6031-6038 (2015).

9. Tang GS, *et al.* Resistive switching with self-rectifying behavior in Cu/SiO_x/Si structure fabricated by plasma-oxidation. *J. Appl. Phys.* **113**, 244502 (2013).

10. Dongyi L, *et al.* Investigations of Conduction Mechanisms of the Self-Rectifying n-Si/HfO_2/Ni RRAM Devices. *IEEE Trans. Electron Devices* **61**, 2294-2301 (2014).

11. Tran XA, *et al.* Self-rectifying and forming-free unipolar HfO_x based-high performance RRAM built by fab-avaialbe materials. In: *2011 IEEE International Electron Devices Meeting (IEDM)* (2011).

12. Wang MJ, Gao S, Zeng F, Song C, Pan F. Unipolar resistive switching with forming-free and self-rectifying effects in Cu/HfO2/n-Si devices. *AIP Adv.* **6**, 025007 (2016).

13. Wookyung S, Hyein L, Hyungsoon S, Wootae L. Investigation of power dissipation for ReRAM in crossbar array architecture. In: *2014 14th Annual Non-Volatile Memory Technology Symposium (NVMTS)* (2014).
